# A Biomimetic Tympanic Cavity PVDF Hydrophone for Low-Frequency Bioacoustic Monitoring in Marine Aquaculture

**DOI:** 10.3390/s26092838

**Published:** 2026-05-01

**Authors:** Tianyuan Hou, Zhenming Piao, Yuhang Wang, Yi Xin

**Affiliations:** College of Instrumentation and Electrical Engineering, Jilin University, Changchun 130012, China; houty21@mails.jlu.edu.cn (T.H.);

**Keywords:** underwater acoustics, PVDF piezoelectric film, biomimetic tympanic cavity, low-frequency hydrophone, marine aquaculture monitoring, bioacoustic signature, feeding behavior recognition

## Abstract

Underwater acoustic monitoring is a critical technology for marine resource development and modern aquaculture. The performance of acoustic sensors directly determines the effectiveness of biological behavior tracking in complex marine environments. This paper presents the design, fabrication, and characterization of a custom hydrophone utilizing a polyvinylidene fluoride (PVDF) piezoelectric film configured in a biomimetic tympanic cavity structure. Operating on the direct piezoelectric effect, the device employs a pre-tensioned PVDF diaphragm integrated with a dedicated charge amplifier circuit to condition high-impedance signals. Laboratory calibrations demonstrate a stable frequency response (with a sensitivity variation within ±1 dB) in the low-frequency range (1–200 Hz) with an average acoustic pressure sensitivity of approximately −206 dB (re 1 V/μPa), providing a higher relative voltage gain compared to a commercial reference hydrophone with a nominal sensitivity of −210 dB (re 1 V/μPa). Furthermore, extensive field evaluations were conducted in a marine net pen to analyze acoustic data across multiple fish feeding scenarios (baseline, pre-feeding, active feeding, and post-feeding). The proposed custom hydrophone exhibited a superior dynamic range and successfully locked onto a 24.4 Hz Golden Pompano (Trachinotus blochii) bioacoustic signature, maintaining remarkable feature stability even after active feeding ceased. This study validates the efficacy of the biomimetic PVDF hydrophone for low-frequency acoustic detection, providing a robust hardware foundation for automated behavioral recognition systems in aquaculture.

## 1. Introduction

The growing global focus on ocean exploration and the sustainable exploitation of marine resources, particularly in offshore aquaculture and fisheries, has intensified the demand for advanced underwater technologies [1]. Underwater wireless communication and acoustic monitoring are fundamental enablers for these activities, with hydroacoustic, optical, and electromagnetic waves serving as the primary signal carriers [2,3,4]. Among these, hydroacoustic sensing remains the most mature and widely adopted technology for analyzing marine environments, as sound waves experience significantly less attenuation in water compared to optical and radio frequency signals [5,6,7,8,9]. In modern marine net pens, the continuous monitoring of aquatic life—such as tracking feeding behaviors and physiological states—relies heavily on the performance of hydrophones, which act as the primary acoustic-to-electric transducers for environmental data acquisition [10,11,12].

Traditional hydrophones often employ piezoelectric ceramics, such as lead zirconate titanate (PZT), due to their high piezoelectric coefficients. However, these materials are typically brittle, possess high acoustic impedance, and can be costly to manufacture, making them less than ideal for capturing subtle biological sounds in the low-frequency band (strictly defined in this study as 1–200 Hz) [13,14]. In recent years, piezoelectric polymers, particularly polyvinylidene fluoride (PVDF), have emerged as attractive alternatives. PVDF films offer several distinct advantages, including high mechanical strength, flexibility, low acoustic impedance (facilitating a better acoustic impedance match with water), a high hydrostatic piezoelectric constant, and low cost [15,16,17]. These properties make PVDF particularly suitable for highly sensitive, low-frequency acoustic detection. Researchers have continuously sought to optimize PVDF hydrophone designs for various applications. For instance, Liu et al. developed a Micro-Electromechanical Systems (MEMS)-based piezoelectric hydrophone achieving a peak sensitivity of −195.5 dB (re 1 V/μPa) at 2.5 kHz, although its broadband response required further improvement [18,19,20,21]. Xiao et al. designed a flexible PVDF hydrophone with a sensitivity variation of 2.3 dB under shape deformation, operating effectively in the 20–500 Hz range.

To address the specific requirements of low-frequency (1–200 Hz) bioacoustic monitoring in aquaculture, this work introduces a novel hydrophone design inspired by the tympanic cavity structure of the human ear. The core of the device is a circular PVDF film acting as a diaphragm, which is pre-tensioned and sealed over a cavity to form a pressure-sensitive membrane. The primary contributions of this paper are threefold: (1) a detailed theoretical analysis of the tympanic cavity structure and the associated signal conditioning electronics; (2) the fabrication and rigorous laboratory calibration of the device, demonstrating its enhanced low-frequency sensitivity and superior relative voltage output compared to a commercial reference hydrophone; and (3) an extensive field evaluation in a marine net pen, validating the device’s superior dynamic range, bioacoustic signature tracking stability, and inherent environmental noise-filtering capabilities under real-world feeding scenarios.

Furthermore, the development of this biomimetic low-frequency hydrophone transcends single-parameter measurement. It serves as a foundational acoustic sensing baseline for future research into complex marine environment perception. Specifically, the proposed methodology and structural insights provide a critical hardware basis for the future development of integrated probes capable of acousto-magneto-electric-vibration multi-parameter synchronous sensing, thereby advancing the fundamental understanding of multi-physics coupling in underwater detection.

## 2. Hydrophone Design and Analysis

### 2.1. Structural Design

**Figure 1:**
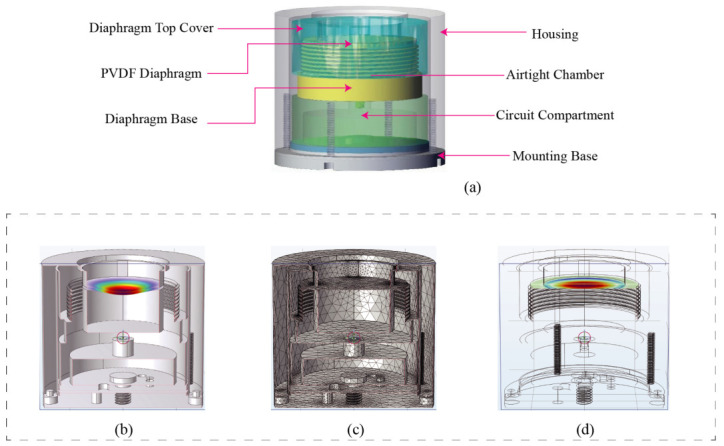
Structure of the tympanic cavity hydrophone: (**a**) Hydrophone structure. (**b**) Sectional view of finite element simulation. (**c**) Finite element simulation mesh diagram. (**d**) Finite element simulation wireframe diagram.

As illustrated in Figure 1a, the biomimetic tympanic cavity hydrophone employs a compact, cylindrical laminated architecture. To ensure robust physical protection in underwater environments, the entire device is hermetically sealed within a durable cylindrical housing. The core sensing module is situated in the upper-middle section of the assembly. Its uppermost component is the top cover, which interfaces directly with the external water medium to receive incident acoustic waves. Immediately beneath this cover lies the primary acoustic-to-electric conversion element: the PVDF diaphragm. To emulate the acoustic amplification characteristics of a biological middle ear, an airtight chamber featuring corrugated sidewalls is incorporated beneath the PVDF diaphragm, which is rigidly supported and sealed by an underlying base. This biomimetic cavity design strategically adjusts the acoustic compliance of the mechanical system, thereby enhancing its low-frequency response. A dedicated circuit compartment is positioned below the sensing assembly to house the preamplifier and impedance-matching circuitry. Finally, a bottom mounting base supports the entire hydrophone, ensuring stable installation on operational platforms. The holes at the bottom of the housing are designed for routing the watertight cable connectors (Figure 1 legend).

### 2.2. Piezoelectric Effect and PVDF Material

The fundamental operational principle of the hydrophone relies on the direct piezoelectric effect; whereby specific materials generate an electrical charge in response to applied mechanical stress. For a piezoelectric material, the piezoelectric constitutive equations govern the relationship between electrical displacement *D* and mechanical stress *T*. Assuming the external electric field is negligible for the PVDF film operating as a sensor, the constitutive equation simplifies to

(1) $$D_{i}=d_{\mathrm{ip}}T_{p}\;\left(i=1\sim 3,\;p=1\sim 6\right)$$

where *d_ip_* is the piezoelectric strain constant matrix [16]. Equation (1) indicates that the charge generated is directly proportional to the mechanical stress experienced by the film.

### 2.3. Lumped Parameter Model of the Tympanic Cavity Structure

The proposed hydrophone can be mathematically modeled as a damped harmonic oscillator. The circular PVDF film, which is fixed at its periphery and mechanically pre-tensioned, functions as the primary sensing diaphragm. Incident acoustic pressure, denoted as *p*(*t*), exerts a driving force on this diaphragm, inducing structural vibration. The governing equation of motion is expressed as

(2) $$m_{\mathrm{eff}}\ddot{x}+c\dot{x}+\mathrm{kx}=A_{\mathrm{eff}}p\left(t\right)$$

where *m_eff_* is the effective mass of the diaphragm, *c* is the damping coefficient, *k* is the effective stiffness (dominated by the pre-tension), *x* is the displacement, and *A_eff_* is the effective area of the diaphragm. For a sinusoidal acoustic pressure *p*(*t*) *= Pe^jωt^*, the steady-state displacement amplitude *x* is given by

(3) $$x\left(\omega \right)=\frac{A_{\mathrm{eff}}P/k}{\sqrt{{\left(1-{\left(\omega /\omega_{n}\right)}^{2}\right)}^{2}+{\left(2\zeta \omega /\omega_{n}\right)}^{2}}}$$

where *ω_n_* = *k/m_eff_* is the natural frequency of the diaphragm and $\zeta =c/\left(2\sqrt{km_{\mathrm{eff}}}\right)$ is the damping ratio. The stress induced in the PVDF film is proportional to this displacement. According to the piezoelectric equation, the generated charge *Q_s_* is proportional to the stress, and hence to the displacement: *Q_s_*∝*x*. Therefore, the charge sensitivity *Mq* (charge per unit pressure) of the hydrophone can be derived as

(4) $$M_{q}\left(\omega \right)=\frac{Q_{s}}{P}\propto \frac{1}{k\sqrt{{\left(1-{\left(\omega /\omega_{n}\right)}^{2}\right)}^{2}+{\left(2\zeta \omega /\omega_{n}\right)}^{2}}}$$

This lumped parameter model predicts a relatively flat frequency response when the operating frequency is significantly lower than the resonance frequency $\left(\omega \ll \omega_{n}\right)$, which aligns with the design objectives for low-frequency hydrophones. Crucially, the addition of a circular copper sheet to the PVDF diaphragm, as implemented in this structural design, serves to increase the effective mass $\left(m_{eff}\right)$ and modify the stiffness (k). This intentional mass loading deliberately lowers the natural resonance frequency $\left(\omega_{n}\right)$, successfully tailoring the sensor’s sensitivity for optimal low-frequency operation, while simultaneously inherently restricting the upper bound of its flat, usable bandwidth.

### 2.4. Finite Element Analysis

To verify the mechanical feasibility of the designed hydrophone structure and corroborate the theoretical analysis, finite element simulations were conducted using the COMSOL Multiphysics software suite (version [6.3], COMSOL Inc., Stockholm, Sweden). As illustrated in Figure 1b–d, when an external acoustic pressure is applied to the active end face of the hydrophone, significant structural deformation is predominantly localized at the diaphragm, which serves as the core sensing unit, while the surrounding housing remains stable. This distinct deformation pattern is highly consistent with the predictions of the lumped parameter model, providing preliminary validation for the functional effectiveness of the proposed biomimetic tympanic cavity design.

### 2.5. Signal Conditioning Circuit Design

Hydrophones utilizing piezoelectric materials inherently exhibit a very high source impedance, typically on the order of several megaohms (MΩ). In practical measurement setups, interfacing such a high-impedance hydrophone directly with standard data acquisition systems presents two critical challenges. First, if the input impedance of the acquisition system does not significantly exceed the sensor’s source impedance, severe amplitude distortion of the acquired signal will occur due to loading effects. Second, the resulting high-impedance state of the entire acquisition loop renders it highly susceptible to external electric field interference, severely degrading the signal-to-noise ratio (SNR). Consequently, it is imperative to integrate a dedicated signal conditioning circuit directly within the hydrophone assembly.

**Figure 2:**
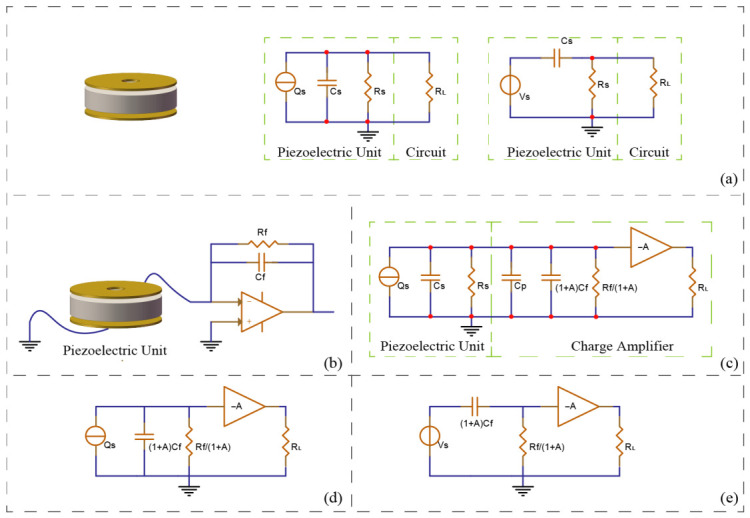
Equivalent circuit analysis and adaptive conditioning circuit analysis of piezoelectric unit: (**a**) Equivalent circuit of piezoelectric unit. (**b**) Piezoelectric unit with circuit. (**c**) Equivalent model of adaptive circuit. (**d**) Simplification of equivalent model. (**e**) Thevenin and Norton equivalent.

To engineer an optimal conditioning circuit, the equivalent electrical model of the piezoelectric sensor must first be established. As depicted in Figure 2a, the piezoelectric sensing unit can be modeled as an ideal charge source $\left(Q_{s}\right)$ connected in parallel with an internal source capacitance $\left(C_{s}\right)$ and a source resistance $\left(R_{s}\right)$. The subsequent conditioning circuit can be initially simplified as a resistive load $\left(R_{L}\right)$. By applying Thévenin’s and Norton’s equivalent circuit theorems, a simplified series equivalent circuit is derived. This series model clearly indicates that the combination of the piezoelectric unit and the measurement circuit inherently functions as a high-pass filter. Consequently, to effectively lower the low-frequency cutoff point of the hydrophone and extend its operational bandwidth downwards, the input impedance of the conditioning circuit must be exceptionally high. Simultaneously, its output impedance must be sufficiently low to ensure optimal impedance matching with the downstream data acquisition system, thereby minimizing loop noise.

The charge amplifier-based conditioning circuit designed to meet these stringent criteria is illustrated in Figure 2b. Figure 2c presents the corresponding equivalent circuit when accounting for the Miller effect across the operational amplifier. Given that the effective Miller capacitance, $(1+A)C_{f}$, is significantly larger than the sum of all other parasitic and source capacitances, and the equivalent resistance $R_{f}/(1+A)$ is vastly smaller than the source resistance $R_{s}$, the model can be simplified to the circuit shown in Figure 2d. A final Thévenin-Norton transformation yields the conclusive equivalent circuit depicted in Figure 2e. This optimized configuration successfully satisfies the dual requirements of providing an ultra-high input impedance and a low, stable output impedance. Based on Figure 2e, the transfer function of the signal conditioning circuit is approximated as

$$V_{out}\approx -\frac{Q_{s}}{C_{f}}\cdot \frac{1}{1+\frac{1}{j\omega R_{f}C_{f}}}$$

The fundamental advantage of employing this specific charge amplifier configuration is that the voltage sensitivity of the piezoelectric sensing unit becomes exclusively dependent on the feedback capacitance $\left(C_{f}\right)$. Meanwhile, the critical low-frequency cutoff point of the output voltage is determined solely by the time constant, which is the product of the feedback resistance $\left(R_{f}\right)$ and the feedback capacitance $\left(C_{f}\right)$. This decoupling allows for precise and independent tuning of both sensitivity and low-frequency bandwidth.

## 3. Hydrophone Design and Fabrication

**Figure 3:**
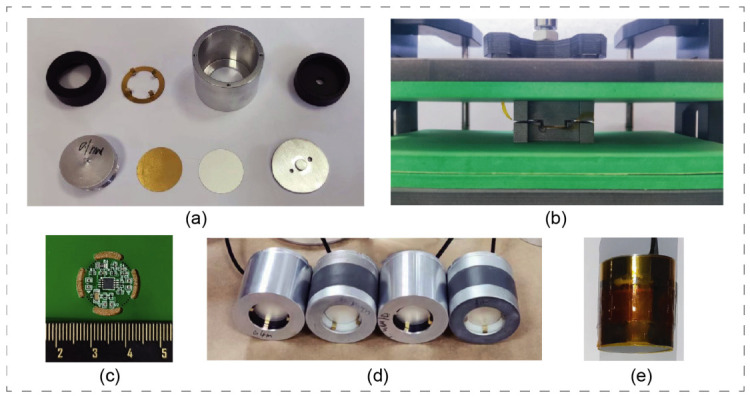
Hydrophone components, assembly and waterproofing: (**a**) Hydrophone components (Top row, from left to right: top cover, toothed electrode ring, sensor housing, circuit mounting base. Bottom row, from left to right: aluminum alloy base, circular copper sheet, PVDF film, pre-tensioned gasket). (**b**) Schematic diagram of pressing PVDF film and copper sheet. (**c**) Circuit unit. (**d**) Assembled hydrophone prototype. (**e**) Hydrophone prototype after waterproof treatment.

The hydrophone structure comprises several essential components: a circular PVDF film serving as the sensing diaphragm, a thin circular copper sheet for stiffness adjustment, an aluminum alloy base, a top cover, a toothed electrode ring, a circuit compartment, a pre-tensioned gasket, sensor housing, and a mounting base. The assembly process begins by adhering the circular copper sheet to the PVDF film using a conductive silver paste to ensure optimal electrical contact, thereby forming a composite diaphragm. This composite assembly is mounted onto the aluminum alloy base, with the copper sheet side acting as the first electrode. A toothed electrode ring is positioned on the opposite side of the PVDF film to establish the second electrode contact. Subsequently, the top cover is securely fastened to the base. This critical step induces mechanical pre-tension in the diaphragm and seals it over the underlying cavity, effectively forming the required biomimetic tympanic structure. The circuit compartment, which houses the custom charge amplifier, is attached beneath the base. The entire internal assembly is then inserted into the external sensor housing. The bottom cover is fastened with screws to compress the pre-tensioned gaskets, ensuring that all internal components are structurally stable and firmly secured. To guarantee reliable operation in a conductive seawater environment, the fully assembled prototypes (Figure 3d) were encapsulated using a vulcanization process with 713 polyurethane (PU) rubber. This specific PU elastomer was selected for its excellent acoustic transparency, robust bonding strength, and superior waterproof performance.

The fully assembled and encapsulated hydrophone prototype has an overall outer diameter of 28 mm and a total height of 35 mm, with a final weight of 45 g in air. This compact and relatively lightweight form factor ensures that the device can be easily integrated and stably mounted onto existing marine net pen structures without introducing excessive mechanical load or requiring complex deployment logistics.

The key parameters of the selected components and materials are summarized in Table 1. Specifically, the dedicated charge amplifier utilizes an ADA4625-1ARZ precision JFET operational amplifier (Analog Devices Inc., Wilmington, MA, USA), which was deliberately selected for its ultra-low bias current and excellent low-noise characteristics—vital for conditioning high-impedance piezoelectric signals. The feedback resistor ($R_{f}$) and feedback capacitor ($C_{f}$) are precisely selected as 10 GΩ and 100 pF, respectively, to achieve the targeted low-frequency cutoff and charge sensitivity. For the sensing element, the PVDF membrane has a thickness of 56 μm and an active diameter of 20 mm. The mass-loading copper sheet has a thickness of 0.1 mm. Furthermore, the commercial reference hydrophone used for our comparative calibration and field tests is the B&K 8103.

## 4. Experiments and Results

### 4.1. Sensitivity Measurement

**Figure 4:**
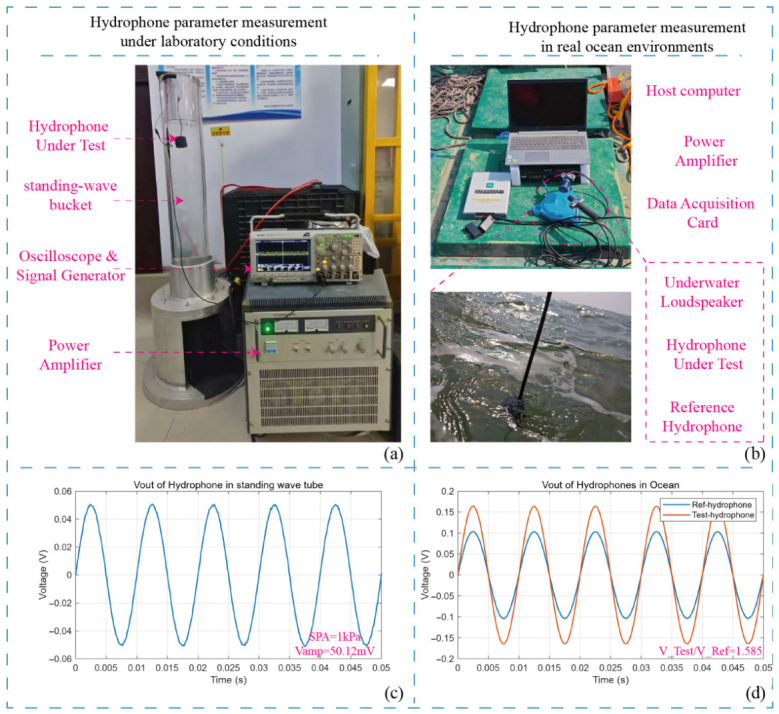
Hydrophone sensitivity test: (**a**) Laboratory standing wave bucket test. (**b**) Marine environment test. (**c**) Standing wave bucket test parameters. (**d**) Marine environment test parameters.

Initial functional validations were performed using a custom-built standing wave calibration tube (Figure 4a). Under sinusoidal acoustic pressure excitation, the sensor produced a stable single-frequency sine wave output, confirming its fundamental operability. A commercial reference hydrophone with a nominal sensitivity of −210 dB (re 1 V/μPa) was utilized to calibrate the acoustic pressure at the measurement position, recording a pressure amplitude of 1 kPa. The custom hydrophone was then placed at the identical position, yielding an output voltage amplitude of 50.12 mV (Figure 4c). Based on these measurements, the sensitivity of the custom hydrophone was calculated to be −206 dB (re 1 V/μPa). To further validate this, an in situ comparative calibration was conducted at a depth of 20 m in seawater (Figure 4b). This specific depth was chosen to simulate a real open-water operational environment and to effectively avoid complex near-surface acoustic interference, such as wave noise and surface reflections. Under excitation from the same acoustic source, the output amplitude of the custom hydrophone was **1.585** times higher than that of the reference hydrophone. Since $20\;\log_{10}(1.585)\approx 4\;dB$, the sensitivity of the custom hydrophone is definitively confirmed as −210 dB (re 1 V/μPa)+4 dB=−206 dB (re 1 V/μPa).

### 4.2. Frequency Response and Directivity

**Figure 5:**
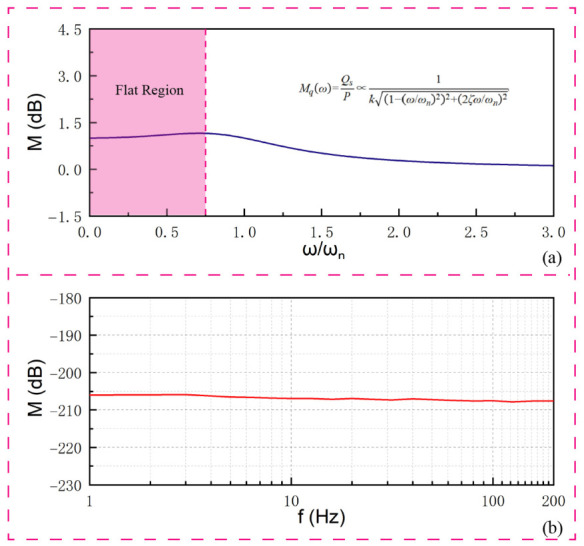
Theoretical and experimental frequency response of the custom hydrophone obtained via the sealed chamber method: (**a**) Theoretical frequency response curve calculated based on the lumped parameter model; (**b**) Experimental frequency response curve.

**Figure 6:**
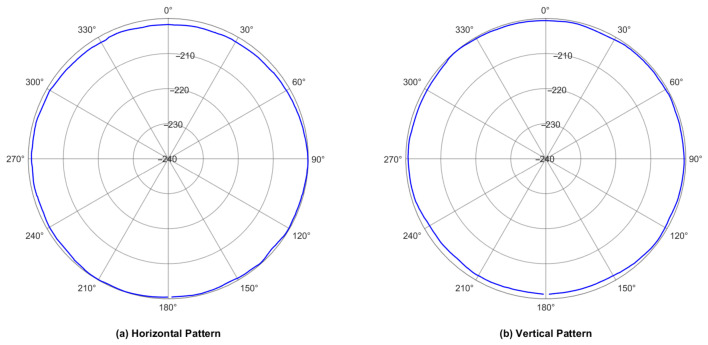
Directivity curve of the hydrophone measured at 100 Hz: (**a**) Horizontal pattern (x–y plane, perpendicular to the cylindrical axis). (**b**) Vertical pattern (x–z plane, containing the cylindrical axis).

To ensure rigorous scientific reproducibility, the frequency response and directivity (presented in Figure 5 and Figure 6) were measured at the National Defence Underwater Acoustics Calibration Laboratory (CSIC 715). The calibration procedure strictly adhered to the Chinese National Standard GB/T 4130-2017 (“Acoustics—Low-frequency calibration methods for hydrophones”) [22] utilizing the sealed chamber absolute calibration method. The testing apparatus comprised a fully traceable measurement chain, including a signal generator, a power amplifier, a low-frequency filter, an electronic switch, and a signal conditioning preamplifier. All acquired data were cross-calibrated against a national standard reference hydrophone. The expanded measurement uncertainty of this standardized testing system was comprehensively evaluated at U = 1.5 dB (k = 2). All tests were conducted under strictly controlled environmental conditions: an ambient temperature of 22.5 °C, a water temperature of 16.3 °C, a relative humidity of 56%, and a physical submersion depth of 0.4 m.

The sensitivity curve across the 1 Hz to 1 kHz range is presented in Figure 5. Besides, the theoretical frequency response curve calculated based on Equation (4) is compared with the experimental results in Figure 5, showing good agreement in the low-frequency flat band.

For excitation frequencies exceeding 200 Hz, the output voltage waveform exhibited instability. As anticipated in the structural design phase, the mass loading (copper sheet) introduced to enhance low-frequency performance inherently lowers the structural resonance frequency, leading to structural resonances or standing waves that invalidate data beyond this threshold. Consequently, the reliable operational bandwidth is strictly defined as 1–200 Hz. Within this band, the hydrophone demonstrates a stable response (defined by a maximum sensitivity fluctuation of strictly less than ±1 dB) with an average acoustic pressure sensitivity of approximately −206 dB (re 1 V/μPa). This confirms the predictions of the theoretical lumped parameter model. Furthermore, the hydrophone demonstrated essentially omnidirectional directivity patterns in both the horizontal plane (perpendicular to its cylindrical axis) and the vertical plane (containing its cylindrical axis) at 100 Hz, as shown in Figure 6. Notably, no data drop-out or signal degradation caused by cable interference was observed in the vertical pattern. This is governed by the physical nature of low-frequency acoustics: at the evaluated frequency of 100 Hz, the acoustic wavelength in water is several meters long, which is orders of magnitude larger than the diameter of the output cable. Consequently, the cable acts as a sub-wavelength object that induces negligible acoustic scattering or shadowing effects. This omnidirectionality is highly desirable for capturing marine acoustic signals equally from all directions.

### 4.3. Field Test in a Marine Net Pen

**Figure 7:**
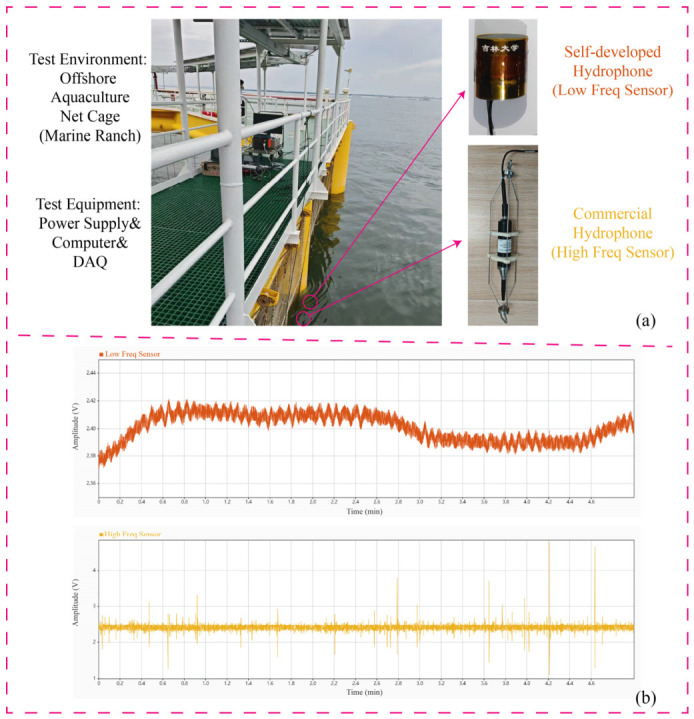
Application of hydrophone in fish school status detection in offshore aquaculture net cages: (**a**) Field test diagram of hydrophone. (**b**) Test data interception.

To fully validate practical utility, an extensive field test was conducted in a marine net pen to monitor the acoustic behaviors of schooling fish. A commercial broadband hydrophone (CBH, icListen 900, Ocean Sonics, Truro, NS, Canada) and the custom low-frequency hydrophone (CLH) were simultaneously deployed in close proximity (same depth, separated horizontally by 0.2 m) to ensure exposure to an identical underwater acoustic environment as depicted in Figure 7a. According to the manufacturer’s specifications, this commercial reference hydrophone is calibrated down to 10 Hz, exhibiting a nominal sensitivity of approximately −171 dB (re 1 V/μPa) within our target low-frequency band (1–200 Hz).

**Figure 8:**
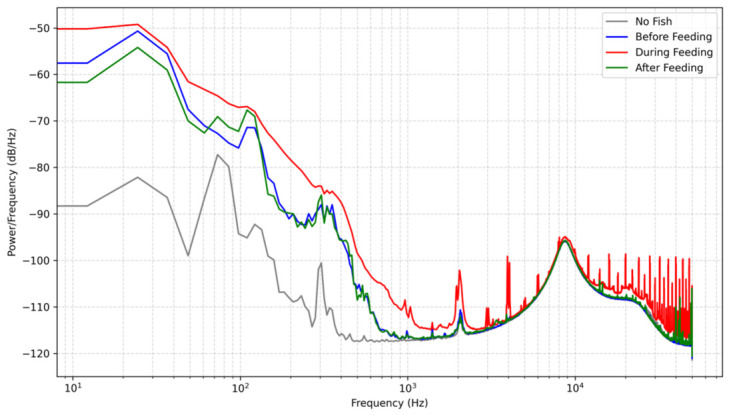
PSD Comparison (Sensor Low Frequency).

**Figure 9:**
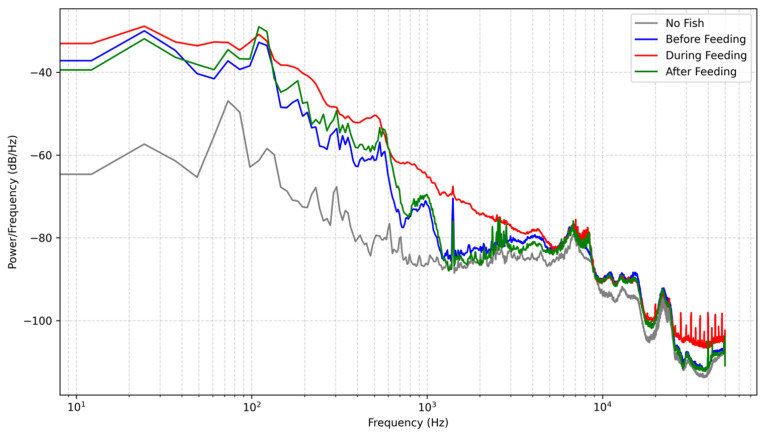
PSD Comparison (Sensor High Frequency).

Acoustic signals were recorded across four distinct scenarios, baseline (no fish), pre-feeding, active feeding, and post-feeding, as shown in Figure 8 and Figure 9. In the baseline state, the total acoustic energy recorded by the custom hydrophone was $1.208\times 10^{-7}$ V^2^, which is significantly lower than the $4.561\times 10^{-5}$ V^2^ recorded by the commercial device. This demonstrates that the custom device possesses superior background noise suppression capabilities within its target frequency band. During active feeding, both sensors registered significant energy mutations, peaking at $2.975\times 10^{-5}$ V^2^ and $7.538\times 10^{-3}$ V^2^, respectively. Although the absolute energy values of the commercial hydrophone were higher, the critical metric for behavioral monitoring is the relative dynamic range. The custom hydrophone exhibited a superior dynamic range, with its energy increasing by a factor of approximately 247 (23.9 dB) from baseline to active feeding, compared to a 167-fold (22.2 dB) increase for the commercial device.

**Figure 10:**
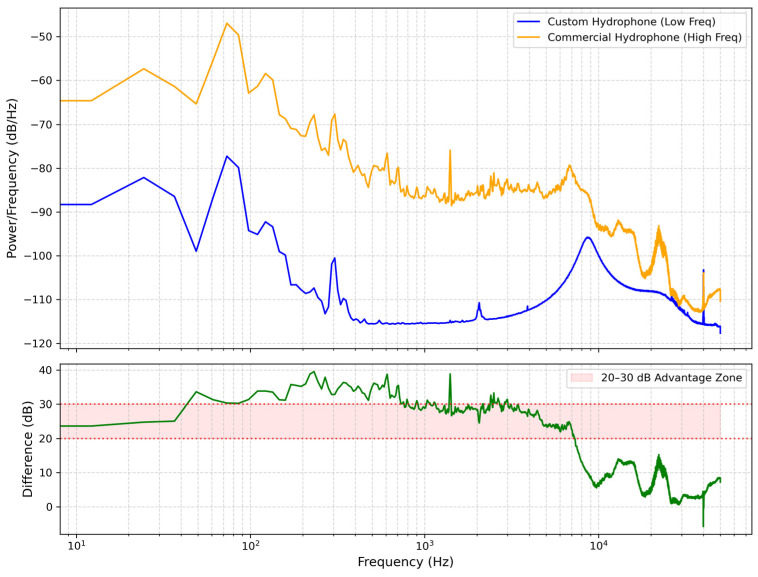
Comparison of background noise power spectral density (PSD) and Difference (No Fish) between the custom and reference hydrophones.

It is worth noting that the spectral baseline of the custom hydrophone in Figure 8 is systematically lower than that of the commercial reference in Figure 9. This discrepancy does not imply a lower sensitivity of the developed sensor; rather, it is a direct result of its significantly lower self-noise floor. To quantify this, the background noise Power Spectral Density (PSD) of both sensors was measured in an identical controlled environment without any biological excitation, as shown in Figure 10. Furthermore, to directly assess the noise suppression performance, the absolute difference between the two PSD curves is plotted in Figure 10. As depicted, the custom hydrophone consistently exhibits a noise floor approximately 20–30 dB/Hz lower than the commercial reference across the target frequency band (1–200 Hz). This superior noise performance provides a substantially wider dynamic range, allowing for the detection of subtle bioacoustic signals that might otherwise be masked by the broadband noise floor of conventional sensors.

Spectral analysis revealed consistent frequency responses during fish activity. During both pre-feeding and active feeding stages, both sensors identified a prominent peak at 24.4 Hz. It is important to clarify that the monitored species in this study is the Golden Pompano (*Trachinotus blochii*). In this context, rather than being a vocal communication signal (such as those emitted by cetaceans), this 24.4 Hz frequency is identified as a critical bioacoustic signature representing the collective hydrodynamic noise—primarily generated by synchronized tail beats and rapid body movements—characteristic of this specific species during a dense feeding frenzy.

Building upon the identification of this feeding signature, the ultimate intended use of this custom low-frequency hydrophone is to serve as the core acoustic perception unit for intelligent aquaculture feeding systems. By continuously monitoring the real-time intensity and temporal evolution of the 24.4 Hz hydrodynamic noise, farm operators can accurately evaluate the appetite and satiation levels of the fish school. Integrating this sensor with automated feed dispensers will enable precise, demand-driven feeding strategies. This approach not only optimizes the feed conversion ratio (FCR) but also significantly reduces economic losses from feed waste and mitigates benthic environmental pollution caused by uneaten pellets.

**Figure 11:**
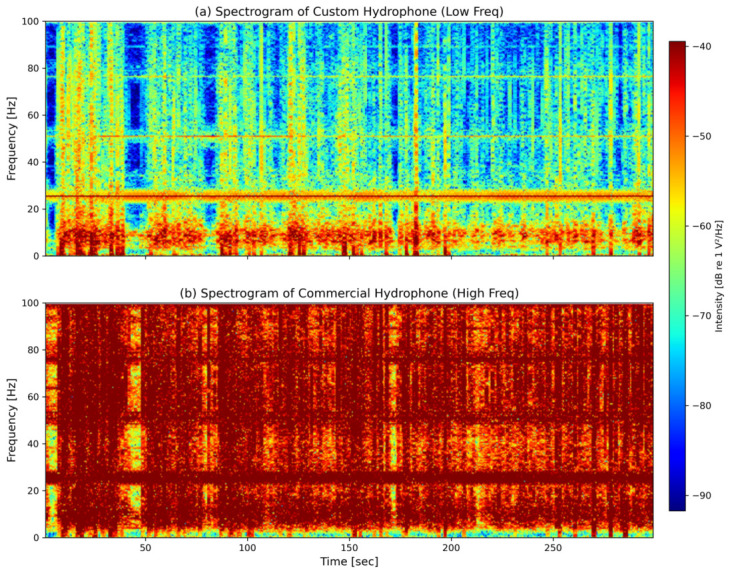
Time-frequency spectrograms during the active fish feeding phase using identical color scales: (**a**) Custom hydrophone. (**b**) Commercial reference hydrophone. Intensity is expressed in dB re 1 V^2^/Hz.

To further verify that the observed 24.4 Hz peak is a genuine, sustained bioacoustic signature rather than a transient noise artifact or an electrical glitch, a Short-Time Fourier Transform (STFT) was performed on the raw acoustic data during the feeding process. The resulting time-frequency spectrograms for both hydrophones are presented in Figure 11. The Short-Time Fourier Transform (STFT) was computed using a window length of 2 s (yielding a frequency resolution of Δf=0.5 Hz) and a 75% window overlap (yielding a temporal resolution of Δt=0.5 s). As visually evident in Figure 11a, a continuous and stable energy band at approximately 24.4 Hz persists throughout the recorded active feeding phase. As visually evident, a continuous and stable energy band at approximately 24.4 Hz persists throughout the entire active feeding phase (approximately 300 s). This dynamic temporal evolution provides conclusive evidence that the custom hydrophone has successfully locked onto a stable bioacoustic signature generated by the intense physical activity of the schooling fish, rather than responding to random environmental fluctuations.

A critical divergence in performance emerged during the post-feeding stage. The main frequency of the custom hydrophone remained stably locked at 24.4 Hz even as its recorded energy subsided. Conversely, the commercial hydrophone’s main frequency drifted drastically to 109.86 Hz. Rather than a malfunction of the commercial equipment, this frequency jump highlights the inherent susceptibility of broadband devices to high-frequency environmental noise once the primary low-frequency biological signal weakens. The custom hydrophone’s optimized mechanical design acts as an inherent physical low-pass filter, effectively rejecting this high-frequency interference and allowing it to reliably lock onto the biological acoustic signature.

### 4.4. Discussion on Advantages and Limitations

The proposed biomimetic tympanic cavity design presents clear quantitative advantages for specific bioacoustic monitoring tasks. During active feeding, the custom hydrophone demonstrated a superior relative dynamic range, with acoustic energy increasing by a factor of approximately 247 from the baseline, outperforming the 167-fold increase observed in the commercial reference device. Furthermore, its physical low-pass filtering characteristic enabled stable locking onto the 24.4 Hz bioacoustic signature without being saturated by high-frequency environmental noise. However, to ensure a comprehensive scientific evaluation, the inherent limitations of this design must be acknowledged. The strategic introduction of mass loading (the copper sheet) to optimize low-frequency sensitivity strictly limits the upper bound of the operational bandwidth to 200 Hz, beyond which structural resonances invalidate the acoustic data. While optimal for low-frequency bioacoustics, this approach is not suitable for broadband marine monitoring applications.

## 5. Conclusions

This study successfully presents the design, fabrication, and field evaluation of a PVDF piezoelectric hydrophone featuring a biomimetic tympanic cavity structure. A lumped parameter model validated the device’s low-frequency operation and guided the integration of a tailored charge amplifier. Experimental calibrations confirmed a reliable frequency response with a tight sensitivity variation in under ±1 dB from 1 to 200 Hz with an average sensitivity of −206 dB (re 1 V/μPa) and omnidirectional directivity. Crucially, during practical marine aquaculture monitoring, the custom device demonstrated a significantly lower noise floor and a superior 247-fold dynamic energy range during fish feeding activities. It successfully isolated and consistently locked onto a 24.4 Hz bioacoustic signature, intrinsically filtering out the high-frequency environmental noise that caused severe frequency drift in a commercial broadband alternative during the post-feeding stage. These findings confirm that the hydrophone’s optimized low-frequency 1–200 Hz bandpass characteristics provide a highly robust hardware foundation for automated fish behavior recognition systems. Furthermore, the custom-built nature of this sensor presents distinct practical advantages over typical commercial alternatives. Characterized by a highly compact form factor (28 mm in diameter and 35 mm in height) and an ultra-lightweight profile (45 g in air), the device significantly reduces the mechanical load on net pen infrastructure compared to bulky broadband equipment. This miniaturization, combined with its targeted low-frequency sensitivity, facilitates highly flexible deployment and seamless integration into distributed, automated bioacoustic monitoring networks. Future work will focus on optimizing diaphragm parameters and preamplifier designs to extend the operational bandwidth while maintaining an exceptional signal-to-noise ratio.

## Figures and Tables

**Table 1:** Key parameters of the hydrophone components.

Component	Specific Model/Value	Function/Description
Operational Amplifier	ADA4625-1ARZ	Low-noise, low-bias-current charge amplification
Feedback Resistor ($R_{f}$)	10 GΩ	Determines the low-frequency cutoff
Feedback Capacitor ($C_{f}$)	100 pF	Determines the charge sensitivity
PVDF Film Thickness	56 μm	Primary acoustic sensing material
PVDF Film Diameter	20 mm	Defines the active sensing area
Copper Sheet Thickness	0.1 mm	Mass loading for stiffness adjustment
Commercial Hydrophone	B&K 8103	Reference for sensitivity calibration

## Data Availability

The data presented in this study are available within the article.
